# Historical review: the German Neurological Society and its honorary members (1952–1982)

**DOI:** 10.1186/s42466-022-00190-z

**Published:** 2022-07-04

**Authors:** Michael Martin, Heiner Fangerau, Axel Karenberg

**Affiliations:** 1 Department of the History, Philosophy, and Ethics of Medicine, Medical Faculty, Heinrich Heine University Düsseldorf, Düsseldorf, Germany; 2 Institute for the History of Medicine and Medical Ethics, University Hospital Cologne, Medical Faculty, University of Cologne, Joseph-Stelzmann-Str. 20, 50931 Cologne, Germany

**Keywords:** Neurology/history, Medicine in National Socialism, Postwar era, Denazification, German Neurological Society, Historical article, Hans Jacob, Gustav Bodechtel, Karl Kleist, Ludwig Guttmann

## Abstract

**Background:**

As part of a larger project commissioned by the German Neurological Society (DGN), this paper focuses on the DGN’s German and Austrian honorary members. In particular, the question of whether former membership in the National Socialist German Workers’ Party (NSDAP) or other Nazi organizations was an obstacle to becoming an honorary member in the years 1952–1982, and whether victims of the Nazi regime were also considered for honorary membership.

**Results:**

From the early 1950s to the early 1980s, the DGN awarded honorary membership to 55 individuals. Of these, 27 were German or Austrian citizens who were physicians during the Nazi era, and 17 of the 27 (63%) were members of the NSDAP, Storm Troopers (SA), or *Schutzstaffel* (SS). In the early postwar period, honorary membership was much less frequently awarded to former Nazi Party members than in the years around 1980. Sir Ludwig Guttmann, the only neurologist forced to emigrate, received his honorary membership in 1971. Brief biographies of Hans Jacob, Gustav Bodechtel, Karl Kleist, and Ludwig Guttmann outline exemplary careers and life histories, in addition to highlighting key issues such as concurrent research on “euthanasia” victims, denazification procedures, forced emigration, and the contemporary mindset in the Federal Republic of Germany.

**Conclusions:**

Apparently, a “Nazi past” did not play a decisive role in the selection process for honorary members within the DGN until at least the 1980s. Aside from Guttmann, no other neuroscientist expelled from Germany was honored. With these practices, the Society marginalized its Jewish colleagues for a second time.

## Background

Sixty-five years after its foundation in 1950, the German Neurological Society (DGN) commissioned and financed a research project on “Neurologists and Neuroscientists in the Nazi Era.” Essential results are summarized in two supplemental volumes, including 19 articles written in German [1, 2].

The aim of this paper is to clarify key aspects of the project for an international readership and to illustrate important points by highlighting a selected group of neurologists. The German-speaking honorary members of the DGN appointed up to 1982, in particular, lend themselves to this purpose because in this group of individuals two fundamental topics can be analyzed: (1) the career paths of neuroscientists during the “Third Reich” and (2) how the DGN Board of Directors have dealt with the Nazi era since the 1950s. Of crucial importance is the question: Did previous membership in the NSDAP (National Socialist German Workers Party), SA (Storm Troopers or “Brownshirts”) and SS (*Schutzstaffel* or “Nazi black shirts”) disqualify an individual’s appointment as an honorary member of the DGN?

In retrospect, this group of individuals had a special status because honorary membership—in contrast to prizes for scientific discoveries and achievements—specifically emphasizes the exemplary character of the honored person and the moral integrity of his or her character. Therefore, historical methodology will assist in determining whether the selected individuals fulfilled these requirements. Did earlier political commitment play any role in the appointment? Were there scientists, clinicians, and practitioners among those honored who had supported the Nazi state and its goals and organizations? If that was the case, how many of them were involved? And would we also find any individuals persecuted by the Nazi regime among the honorary members?

The present essay attempts to answer such questions. The first section deals with sources and methodology as well as inclusion and exclusion criteria. In the second section, the focus is on the relationship of DGN honorary members to the Nazi regime and, specifically, possible membership in the NSDAP, SA, and SS. In addition, four biograms describe forced migration, denazification procedures, and professional advancement in the postwar period. In the final chapter, the main results are discussed, and meaningful conclusions are drawn with a bearing on the present.

## Material and methods

The current DGN website lists 90 honorary members, including those from a precursor organization [3]. No appointments were made in 1950/1951, and from the mid-1980s onward, appointed members, due to their age, would not have been involved in medical activity during the Nazi era; therefore, we concentrated on the period 1952–1982, during which 55 individuals were listed as honorary members. Since only German and (after the “Anschluss” in 1938) Austrian citizens were eligible to become members of the NSDAP, SA, SS, and other Nazi organizations, all honorary members with a citizenship other than German or Austrian were excluded from further investigation. This exclusion criterion reduced the number to 28 honorary appointments. An exception within the sample was Sir Ludwig Guttmann, who was born in the German Reich, educated at German universities and clinics, and acquired British citizenship after his forced emigration (see below). With the focus on the DGN in West Germany, doctors who continued their careers in the German Democratic Republic after the war were also excluded [4]. Other scientists included worked in related neurological fields, such as psychiatry, neurosurgery, and neurogenetics. Also considered were neurologists who, in addition to being awarded honorary membership, served as Chairman or honorary Chairman of the DGN [5].

The group of individuals selected using the above criteria consisted of 24 Germans, three Austrians, and one naturalized Briton (Table 1). Source material from the Federal Archive in Berlin-Lichterfelde (formerly the Berlin Document Center) was evaluated as extensively as possible, particularly regarding NSDAP, SA, and SS membership. The research also covered numerous state, regional, and university archives. Unfortunately, the DGN does not have its own collection of documents from the period of interest examined in this report, except for minutes of the meetings of the Executive and Advisory Board from 1979 to 1986. As a final step, we analyzed relevant publications of the 28 honorary members and published biographical sources, including scientific appreciations or obituaries, and pertinent secondary literature to complete the available sources of information.

**Table 1:** Honorary members of the German Neurological Society (DGN) (1952–1982) and their membership in NSDAP, SA, and SS

No.	Name	Biographical data	Age as of 30 January, 1933	Appointment as honorary member	Acting DGN president	NSDAP membership	SA membership	SS membership
1	Albrecht Bethe	25.4.1872–19.10.1954	60	1952	Pette	–	–	–
2	Richard Henneberg	22.12.1868–25.1.1962	64	1952	Pette	–	–	–
3	Ferdinand Hochstetter	5.2.1861–10.11.1954	71	1952	Pette	–	–	–
4	Ludwig Robert Müller	26.4.1870–8.9.1962	62	1952	Pette	–	–	–
5	Oskar Vogt	6.4.1870–21.7.1959	62	1952	Pette	–	–	–
6	Cécile Vogt	27.3.1875–4.5.1962	57	1952	Pette	–	–	–
7	Viktor von Weizsäcker	21.4.1886–9.1.1957	46	1952	Pette	–	–	–
8	Karl Kleist	31.1.1879–26.12.1960	53	1954	Schaltenbrand	+ 1940	–	–
9	Martin Reichardt	17.8.1874–23.12.1966	58	1954	Schaltenbrand	+ 1937	–	+ 1933sm
10	Siegfried Schönborn	31.12.1874–11.3.1966	58	1956	Vogel	+ 1937	?	?
11	Ludwig Guttmann	3.7.1899–18.3.1980	33	1971	Bauer	–	–	–
12	Gustav Bodechtel	12.3.189–10.7.1978	33	1976	Behrend	+ 1937	+ 1933	–
13	Wilhelm Tönnis	16.6.1989–12.9.1978	34	1976	Behrend	+ 1937	+ 1934 SH	+ 1933sm
14	Richard Jung	27.6.1911–25.6.1986	21	1978	Schliack	–	+ 1934	–
15	Paul Vogel	15.4.1900–2.9.1979	32	1978	Schliack	+ 1937	–	–
16	Klaus Joachim Zülch	11.4.1910–2.12.1988	22	1978	Schliack	+ 1937	+ 1933	–
17	Eberhard Bay	12.12.1908–1.5.1989	24	1979	Mertens	+ 1937	–	–
18	Hans-Robert Müller	4.3.1901–4.12.1981	31	1979	Mertens	+ 1937	–	–
19	Heinrich Kalm	2.3.1915–25.12.1981	17	1980	Mertens	–	–	–
20	Herta Lange	9.7.1907–16.11.2005	25	1980	Mertens	–	–	–
21	Johannes Hirschmann	7.3.1910–30.3.1991	22	1981	Seitz	+ 1937	+ 1933	–
22	Hans Kuhlendahl	2.7.1910–24.2.1992	22	1981	Seitz	+ 1937	+ wd	–
23	Karl-Ernst Pass	?–?	?	1981	Seitz	–	–	–
24	Helmut J. Bauer	31.3.1914–16.1.2008	18	1982	Seitz	–	–	1941
25	Peter Emil Becker	23.11.1908–7.10.2000	24	1982	Seitz	+ 1938	+ 1933	–
26	Walther Birkmayer	15.5.1910–10.12.1996	22	1982	Seitz	+ 1933–1939	+ 1932–1936	+ 1936–1939
27	Hans Jacob	13.11.1907–1997	25	1982	Seitz	+ 1937	+ 1934	–
28	Franz Seitelberger	4.12.1916–2.11.2007	16	1982	Seitz	+ appl	–	+ 1938

+ member, – no member, *wd* without date, *sm* supporting member, *appl* applicant, *SH* Stahlhelm (paramilitary organization)

## Results

### Age and gender of honorary members

The average age of the 28 neuroscientists, who later received an honorary membership, at the time of Hitler’s seizure of power was 37 years. Table 1 illustrates that there were two clearly separated timeframes within the selected study period covering almost all appointments relevant for this investigation: the years 1952–1956 (Cluster 1), and the period from 1976 to 1982 (Cluster 2). In between were the two decades in which the DGN followed a clear internationalization strategy regarding its appointments and did not award honorary memberships to German or Austrian scientists.

Within Cluster 1, the average age on January 30, 1933, was 58 years. The oldest honorary member was the Viennese neuroanatomist Ferdinand Hochstetter at 64 years of age, and the youngest was the neurologist von Viktor von Weizsäcker, aged 46. Many of the honored individuals had already attained a senior position and a full professorship by the time Hitler came to power: Hochstetter [6] and Albrecht Bethe [7] as directors of a preclinical institute; Ludwig R. Müller [8], Karl Kleist, and Martin Reichardt [9] as heads of a university clinic; Oskar Vogt as head of a non-university research institute [10, 11]; and Cécile Vogt and von Weizsäcker [12] as department heads. Siegfried Schönborn was employed as a chief physician [13] and Richard Henneberg as a resident neurologist and active researcher [14]. Overall, these individuals were between 66 and 91 years of age at the time of their appointment as honorary members of the newly founded DGN.

The situation is different in Cluster 2. The neurosurgeon Wilhelm Tönnis, born in 1898, was the oldest honorary member, and Franz Seitelberger, born in 1916, was the youngest, and part of a group of honorees almost one generation younger. As a result, this group of individuals was on average only 24 years old when Hitler seized power, and in 1933 most of them were still studying medicine or working as assistants. However, it was not until the sixth or seventh decade of their lives that they received honorary membership in the DGN.

Cécile Vogt and Herta Lange were the only women in the group of honorary members. The former published internationally acclaimed research on cyto- and myeloarchitectonics of the central nervous system at the Kaiser Wilhelm Institute for Brain Research (KWIBR) in Berlin (headed by her husband) and by 1950 was nominated for the Nobel Prize nine times [15, 16]. The latter made a name for herself primarily as a child neurologist and psychiatrist [17]. To date, no other female honorary members of the DGN have been added to the current roster.

### Political activities 1933–1945

Among the 27 German or Austrian honorary members, membership in the NSDAP could be established for only 14 persons, with one neurologist found to be an aspirant candidate [18]. At this point, a distinction according to the time period of the honorary memberships proved to be revealing: while only 3 out of 10 honors awarded in the 1950s went to former NSDAP members (Kleist 1954, Reichardt 1954, and Schönborn 1956), of the 17 honored in the 1970s and 1980s, as many as 12 had applied for a Party membership book and 11 had received it. In the majority of cases, 1937 was determined as the year of entry. Only Birkmayer joined the Party, which was still illegal in Austria at the time, as a student in 1933, but was expelled in 1939, as he lacked a “certificate of Aryan descent” (*Ariernachweis*) [19]. The neurogeneticist Peter Emil Becker [20] and the Frankfurt professor Kleist became Party members in 1938 and 1940.

Nine subsequent honorary members belonged to the SA, one of whom was an earlier member of the so-called paramilitary Steel Helmet (*Stahlhelm*) which merged with the SA in 1934. Only the years 1932–1934 are registered as entry dates. Three individuals belonged to the SS, and two registered as so-called supporting members. In addition, many of them were members of other Nazi organizations and associations, primarily the National Socialist German Doctors̕ League (NSDÄB), the National Socialist German Lecturer Association (NSLB) and the Nazi Students’ Association [21]. In summary, 17 out of 27 (nearly two thirds) of the German and Austrian honorary members of the DGN appointed between 1952 and 1982 were at least formally associated with the “Third Reich” through the NSDAP or other Nazi organizations. The extent of an ideological affinity or overlap in health or research policy between scientists and the Nazi state was explored by focusing on four prototypical career neuroscientists from that period.

### Professional careers of honorary members

#### Hans Jacob: a beneficiary of “concurrent research”

Historians agree that German neuroscientists were involved in various “euthanasia” activities, in that they utilized the brains of murdered patients to increase their medical knowledge. Julius Hallervorden (1882–1965) [22] and Hugo Spatz (1888–1969) [23] have been the primary focus of interest to date; however, the participation of another DGN honorary member in this “concurrent research” has only recently become known. Hans Jacob studied medicine in Dresden, Kiel, Vienna, Munich, and Leipzig. In Leipzig he met the senior physician Hans Bürger-Prinz, who would play an important role in his future career. Bürger-Prinz (1897–1976) was a member of the NSDAP and NSDÄB and was appointed head of the Psychiatric and Neurological Clinic at the University Hospital Hamburg-Eppendorf in 1936. One year later, he appointed Hans Jacob as head of the Clinic’s Brain Anatomy Laboratory [24].

Jacob’s entry into the NSDAP and the NSDÄB in May 1937 could be related to his new appointment in Hamburg, since candidates for such positions were expected to be members of National Socialist organizations. However, Jacob may have applied for admission much earlier, as the NSDAP instituted a ban on admissions starting in May 1933, and older applications from this period were dated May 1, 1937. The fact that Jacob had already joined the SA in 1934 and held the rank of squad leader (*Rottenführer*) is evidence of a positive attitude toward National Socialism that he’d held for some time [25].

Jacob had studied neuropathology since 1930 [26]. His quest for “research materials” coincided with the beginning of the National Socialist “children’s euthanasia” program in 1939 and the introduction of specialized “children’s departments.” These included wards in mental hospitals (psychiatric asylums, in particular) and nursing homes, where research in euthanasia, as conceptualized by the Nazis, was carried out. Ultimately, thousands of physically or mentally disabled children and young people were killed [27, 28].

It was in this context that Jacob encountered one of these specialized “children's departments”, the Langenhorn Asylum and Nursing Home, and its director Friedrich Knigge (1900–1947): A study by Burlon noted that “some dissection protocols have been preserved, they bear Knigge’s signature and provide information about the cause of death and the whereabouts of the brain. Knigge sent the brains to Jacob in the Psychiatric and Neurological Clinic of the University Hospital Eppendorf. Knigge testified that Hans Bürger-Prinz had entrusted his prosecutor Hans Jacob with the neuropathological examinations” (all translations by AK, HF and MM) [29].

Human brains also came from another children’s department at the State Asylum and Nursing Home in Lüneburg. The number of organs entering Jacob’s laboratory in Hamburg increased from 175 in 1939 to 224 in 1940 and to 300 in 1942 [25]. Jacobs’ great interest in brains is evident from a letter to Knigge dated February 11, 1942: “Dear Dr. Knigge! Thank you again for kindly letting me have the brain. However, I was only able to get one case. As your pathology nurse informed Mr. Moebert, the other two cases mentioned have not been dissected. (…) With best wishes and awaiting further brains! Sincerely yours, Dr. Jacob” [25, 29].

**Fig. 1:**
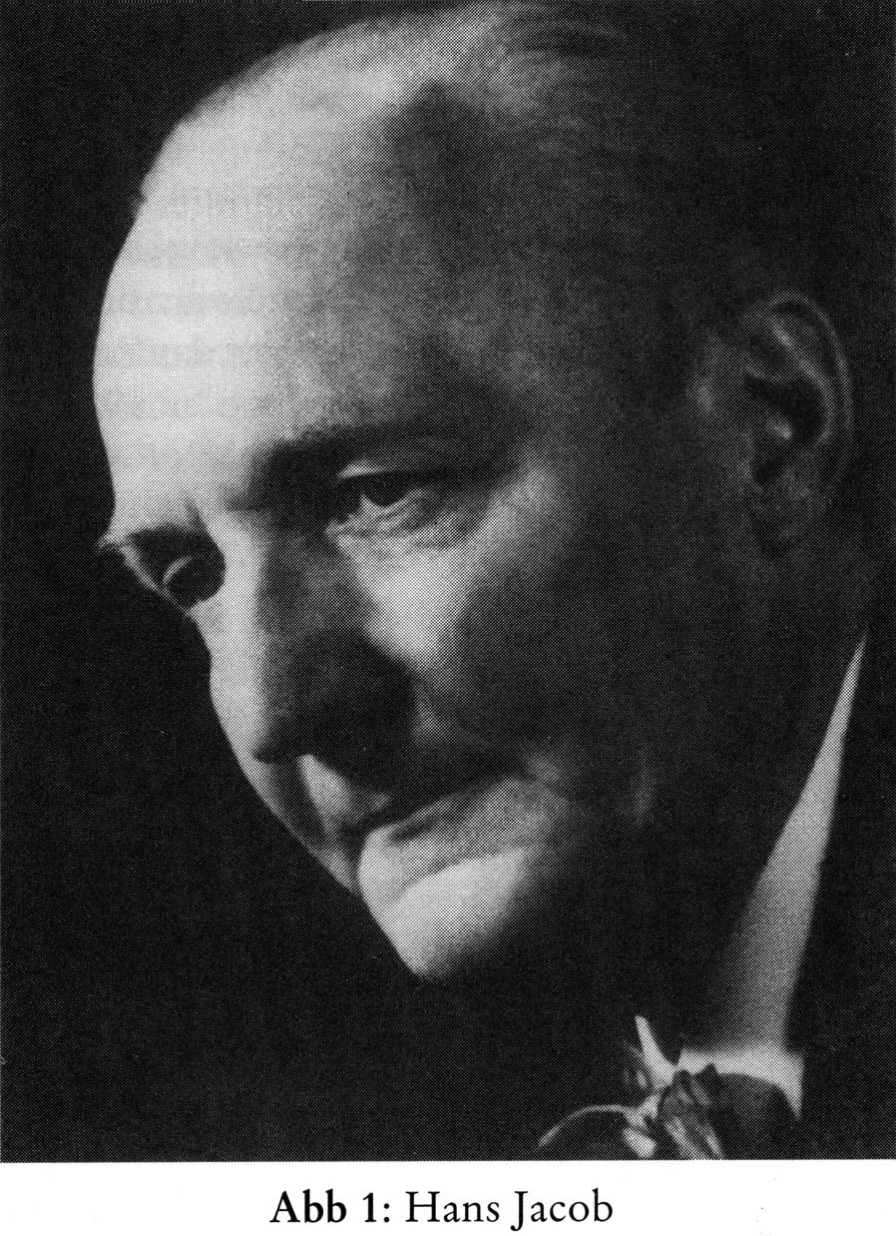
Hans Jacob. Undated photography, once hanging in the Psychiatric and Neurological Clinic of the University of Marburg. With permission of Professor Hans-Dieter Mennel

It is no longer possible to verify exactly how many brains of the victims from children’s departments Jacob dissected. Cautious estimates amount to around 42 cases [25]. At least one postwar publication is based on specimens from two patients killed in Lüneburg [30]. In his denazification trial, Jacob was classified as “exonerated” and was allowed to continue his career without any difficulties, initially as head of the neurological department at the General Hospital in Hamburg-Altona. In 1959 he accepted the offer of a full professorship in Marburg [26]. In 1982 he was appointed an honorary member of the DGN (Fig. 1). Exactly what the individuals in charge at the DGN knew—or wanted to know—about Jacob’s past remains an open question.

Regarding Jacob’s involvement in research on the brains of euthanasia victims, Zeidman notes that: “No documents were found that might confirm whether or not Jacob knew the tainted source of the specimens delivered to him” [25]. Nevertheless, it is scarcely imaginable that such a venerated scientist at the height of his profession and with his connections (Julius Hallervorden, for exampled) could have been completely clueless about the origins of his own research specimens. Jacob himself makes no comment on this, not even on his stance to euthanasia performed in this context.

Franz Seitelberger, honorary member of the DGN in 1982, benefitted in a similar way from such concurrent research, but only after the war. In the 1950s, he used brain specimens that could be traced back to the killing centers Am Spiegelgrund (Vienna) and Brandenburg-Görden [31–33].

#### Gustav Bodechtel: a typical denazification case

Immediately after the end of the war, legal persecution and so-called denazification began, which all active neurologists in occupied Germany were obliged to undergo [34]. In 1946, the proceedings passed into German hands and included the classification of five categories of offense: (I) Major Offenders, (II) Offenders, (III) Lesser Offenders, (IV) Followers, and (V) Persons Exonerated. Many of the defendants appealed against their supposedly “false” categorization, often successfully. The exemplary case of Gustav Bodechtel, honorary member of the DGN since 1976, depicts the crux of denazification.

On April 3, 1946, Bodechtel was dismissed from his position as full professor and director of the II. Medical Clinic of the Medical Academy in Düsseldorf, primarily due to his membership in the SA starting in 1933. In addition, he had been a member of the NSDAP since May 1, 1937 [35]. In the questionnaire he completed as part of denazification procedure, he mentioned additional memberships: National Socialist German Doctors̕ League (1935–1945), Reich Colonial League (1942–1945), and State Air Protection Corps (1941–1945) [36]. Thus, his affinity with National Socialism was indisputable.

Bodechtel filed an objection against his dismissal, stating: “From 1932 I was a lecturer at the Medical Faculty of Erlangen. Hence, my entry into the SA in August 1933 was the result of being put under pressure; that is, I was repeatedly warned that I could not continue my university career unless I joined the NSDAP or one of its branches.” On the advice of colleagues, he finally accepted the “quandary,” given that his career was being “compromised.” Regarding his function in the SA, he explained: “My activity in the SA was extremely apolitical; I only carried out medical screenings, and my appointment to Medical Squad Commander in 1938 was only made to bring my rank in the Sanitary Department of the SA in line with my civil position as a professor of medicine.” Regarding his Party membership he added: “My entry into the NSDAP in May 1937 took place without my involvement, when the SA members were incorporated into the Party” [36]. With this statement Bodechtel followed a familiar strategy, documented again and again in the records of other trials. The circumstances of the time, so the argument went, left certain individuals with no other option but to join the Party or another Nazi organization and, above all, one did “only what was necessary.” In addition, the files of numerous DGN honorary members include claims that their membership in the NSDAP occurred without their knowledge. These assertions have since been proven wrong by historians.

Bodechtel also stressed his international connections: “Through friendly relations with many foreign researchers, I could effectively help colleagues emigrate after 1933.” He named witnesses who could back his statements and enclosed affidavits signed by former colleagues. These attestations, popularly called “persil” certificates (in that the document “whitewashed” the possible guilt of its holder) (*Persilscheine*) [37], fully confirmed what Bodechtel had attested. His most prominent advocate was the nestor of German neurology, the former Hamburg professor Max Nonne [38].

Only a week later the dismissal was annulled by the Denazification Committee in Düsseldorf. Bodechtel was admittedly “culpable of one thing,” namely his SA membership. In his personal file the denazification protocol states: “But due to his resolutely anti-Nazi attitude, and neither a militarist nor hostile to the goals of the Allies, he actually should be regarded as the least culpable.” One member of the Committee agreed with Bodechtel about having been forced to join the SA, and his promotion to “Squad Commander of the SA was considered to be a scientific recognition rather than a political one (…) Despite his position in the SA, he must be rated as a non-active National Socialist” [38]. Bodechtel was thus classified in category IV “Followers.”

**Fig. 2:**
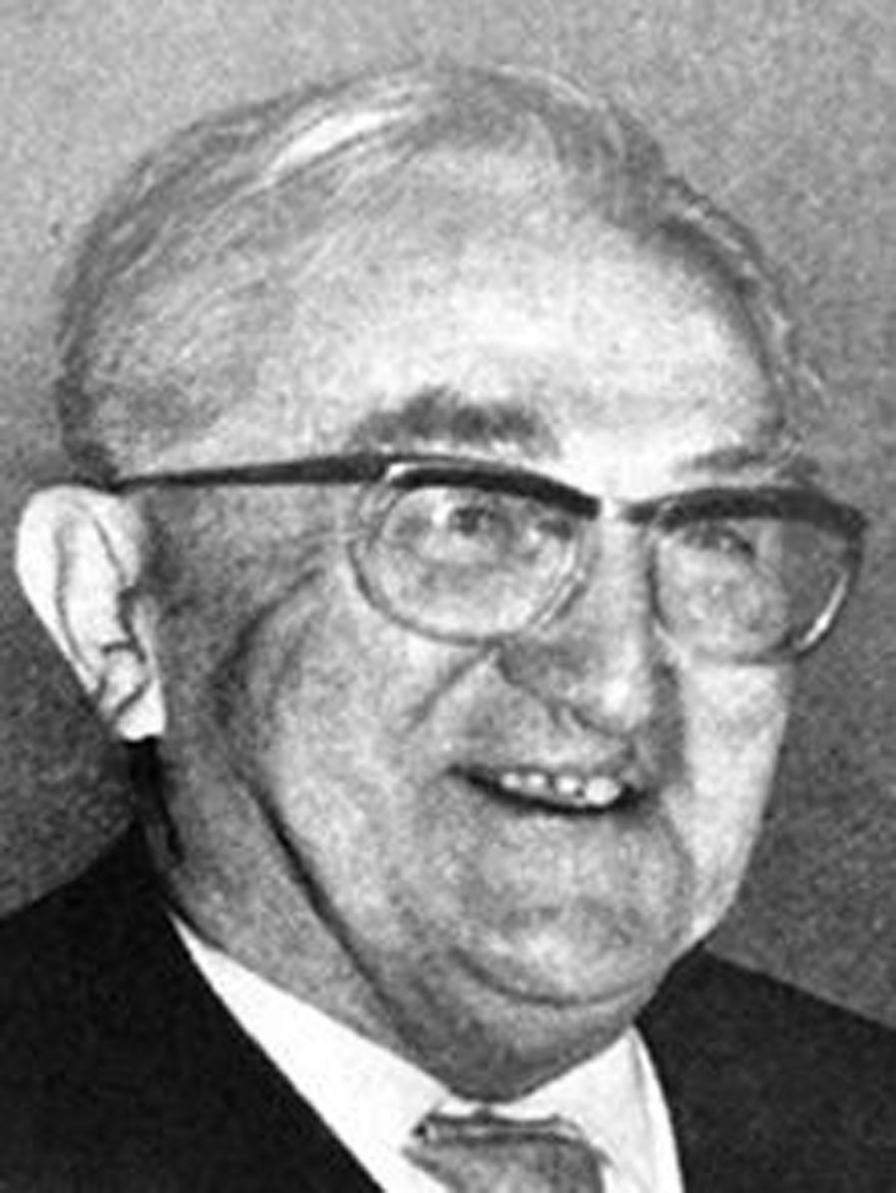
Gustav Bodechtel. Undated photography, photographer unknown. *Source*: Steinmetz, H. History of neurology in Germany. https://dgn.org/uber-uns/geschichte/history-of-neurology-in-germany/

The expeditious course of his trial was also due to the lack of physicians in the immediate postwar period. Accordingly, his employer, the Düsseldorf Medical Academy, had written a letter to the commander in charge of the U.S. Army: “Prof. Dr. Bodechtel is indispensable for tending to neurologically ill patients with traumatic brain and nerve lesions and thus of vital importance.” The Lord Mayor stated “that the absence of Dr. B. weighed on the medical care of the population” [38]. Eventually, the Headquarters’ Military Government officially confirmed that: “Prof. Bodechtel is to be reinstated to his position at the Municipal Hospital and the Medical Academy as of September 10, 1946” [38]. He was able to pursue his career as a full professor in Düsseldorf and Munich and is today considered one of the most renowned German neurologists of the postwar period (Fig. 2).

Like Bodechtel, the later honorary members Richard Jung [39], Klaus Joachim Zülch [40], and Walther Birkmayer [19] were dismissed due to their membership in the SA. As a result of his activities in the National Socialist Flying Corps (NSFK) the Hamburg neurologist Hans-Robert Müller lost his job [41]. None of these men accepted their dismissal and subsequently launched an appeal. After the denazification process ended, all four pursued their professional careers.

#### Karl Kleist: critical distance and cooperation

**Fig. 3:**
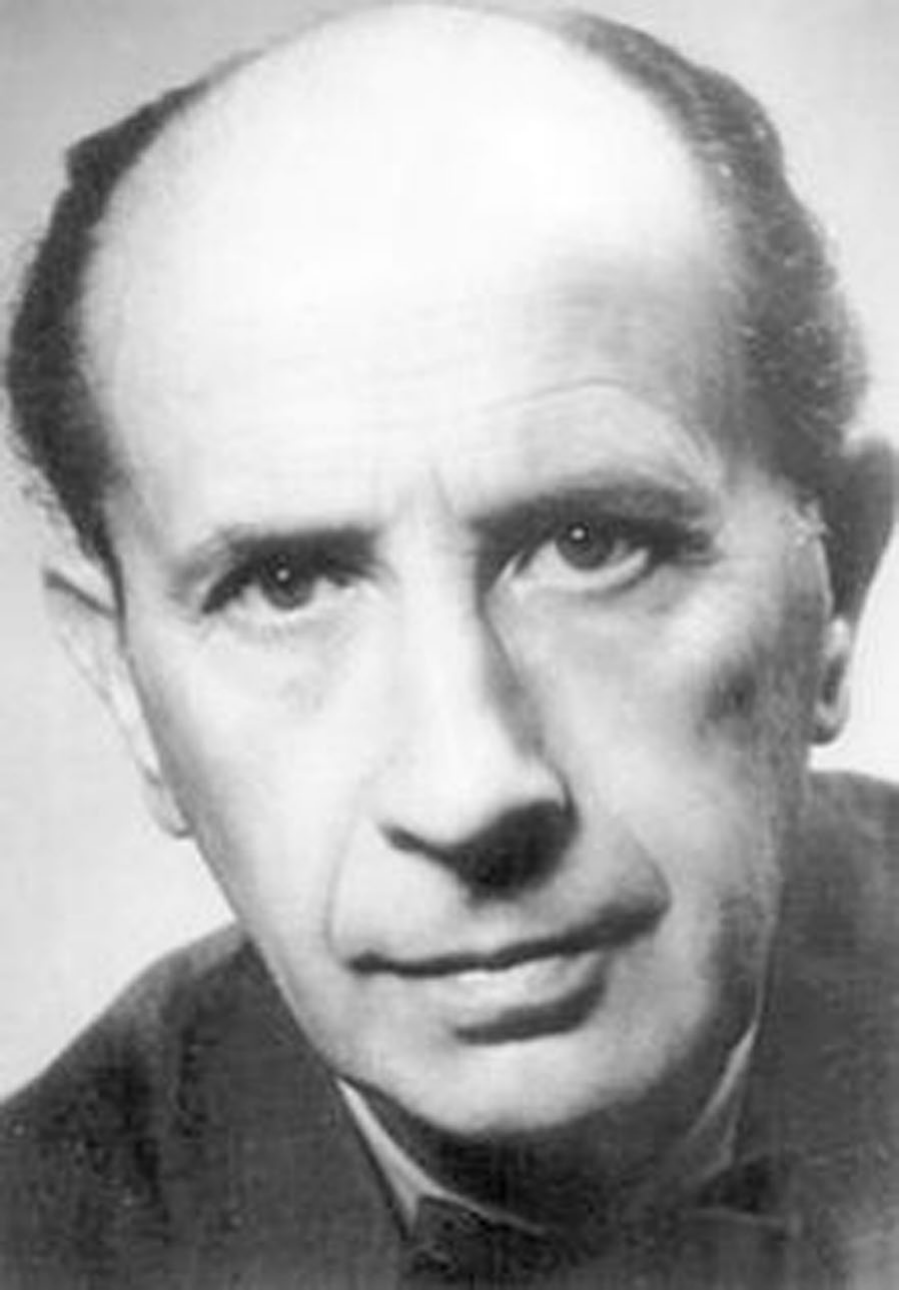
Karl Kleist. Undated photography. *Source*: Leonhard K. (1961). Karl Kleist zum Gedächtnis 31. Januar 1879–1926. Dezember 1960. *Archiv für Psychiatrie und Nervenkrankheiten Zeitschrift für die Gesamte Neurologie Psychiatrie, 202*, 451–456. https://doi.org/10.1007/BF00342107

Karl Kleist, appointed as an honorary member in 1954, had his own way of dealing with National Socialism (Fig. 3). Alexander Mitscherlich, an observer of the Nuremberg Doctors’ Trial, highlighted Kleist as one of the very few among the medical professionals who defied National Socialist views [42].

To call him an opponent of the National Socialists, however, would be misguided. Having worked in Halle, Erlangen, and Rostock, Kleist became a full professor of psychiatry and neurology at the University of Frankfurt am Main in 1920. He had numerous “non-Aryan” employees whom he supported [43]. Because Kleist continued to treat Jewish patients, the Frankfurt psychiatric department was also called the “Jewish Clinic” (*Judenklinik*) [44]. Kleist is said to have refused the transfer of patients to the nearby psychiatric institution at Eichberg, known for its involvement in the “children’s euthanasia” program. It was from there, that young patients were transferred to the Hadamar killing center. The head of the administration in Eichberg called it “an act of deliberate resistance” [45]. According to the neurologist and geneticist Gerhard Koch, it was also tacitly understood that at “the Kleist Clinic” diagnoses of schizophrenia and manic depression were mostly avoided during the years of the Third Reich for fear of forced sterilization [46, 47].

On the other hand, Kleist worked as an expert witness for National Socialist hereditary health courts. He was critical of sterilization and forced sterilization, in particular, which he considered to be untenable from a scientific point of view [44]. This attitude earned him much criticism, especially concerning his work as an expert witness. In an internal letter from 1935, the influential Nazi racial hygienist and functionary Ernst Rüdin (1874–1952) stated (according to Schmuhl’s findings) that Kleist’s ideas “on the whole conform with the sterilization law, yet in clinical matters he is often alone in his convictions, and many people will not agree with his conclusions about sterilization based on his particular clinical views” (…) [48].

This distancing from the official Nazi line in terms of scientific assessments did not prevent Kleist from holding various positions in the state and the Party. Thus, he was not only an expert witness at the Appellate Hereditary Health Court in Frankfurt am Main, but also a member of the NSDAP as of 1940, and of the National Socialist German Doctors̕ League since 1942. He worked as an Army (*Wehrmacht*) medical officer and as a military psychiatrist in Military District IX in Frankfurt [49]. From 1936 to 1941, he was also a member of the Board of Trustees of the KWIBR—a Board not known for distancing themselves from the Nazi regime [50].

Kleist’s behavior, which wavered between detachment and cooperation, appears contradictory. Regarding this issue, he made the following comment to his former Jewish colleague Leo Alexander (1905–1985), who shortly after the war interviewed scientists at the behest of the Americans: “I never thought much of the sterilization law. Its justification in the manic-depressive group was always questionable, to say the least. Apart from the fact that the way the whole setup was organized implied an unnecessary expenditure, it was medically unsound because it dismissed the patient as a human being and discredited his or her entire family. I had an extensive practice as an expert in sterilization cases, especially in cases that were appealed to the higher courts of hereditary health (*Erbgesundheitsobergerichte*), and I have always tried to protect patients from sterilization. The only cases in which I regarded sterilization as justified were in Huntington’s chorea, severe mental deficiency, and severe cases of epilepsy” [51].

The assessment of this position remains controversial to this day. Some see Kleist’s behavior as a form of resistance, which was within the realm of possibility, at least as a means of undermining National Socialist standards. Others view him as an example of the allegedly “apolitical” scientist: “Kleist appears to have been a conservative German scientist whose main interest was science and caring for his patients, and who did not oppose the criminal regime of National Socialism beyond his narrow professional field” [52]. After the war, Kleist was briefly suspended, but then allowed to return to his old positions, in which he remained until his retirement in 1950.

An ambivalent attitude toward the Nazi regime and its health policy can also be found among other honorary members. Viktor von Weizsäcker (1886–1957) shunned entry into central Nazi organizations and was considered “politically unreliable” in the judgment of colleagues loyal to the system. On the other hand, he supported, to a certain degree, the Law for the Prevention of Hereditarily Defective Offspring (*Gesetz zur Verhütung erbkranken Nachwuchses*), used relevant Nazi terminology in lectures and publications from 1933 to 1935, and coined the term “doctrine of annihilation” (*Vernichtungslehre*) [53]. Despite their commitment to Jewish scientists and fellow citizens, Oskar and Cécile Vogt also tried to present their research as eugenically useful and to make it at least partially compatible with Nazi health policy [54].

#### Sir Ludwig Guttmann (1899–1980): a victim of the National Socialist era as honorary member

**Fig. 4:**
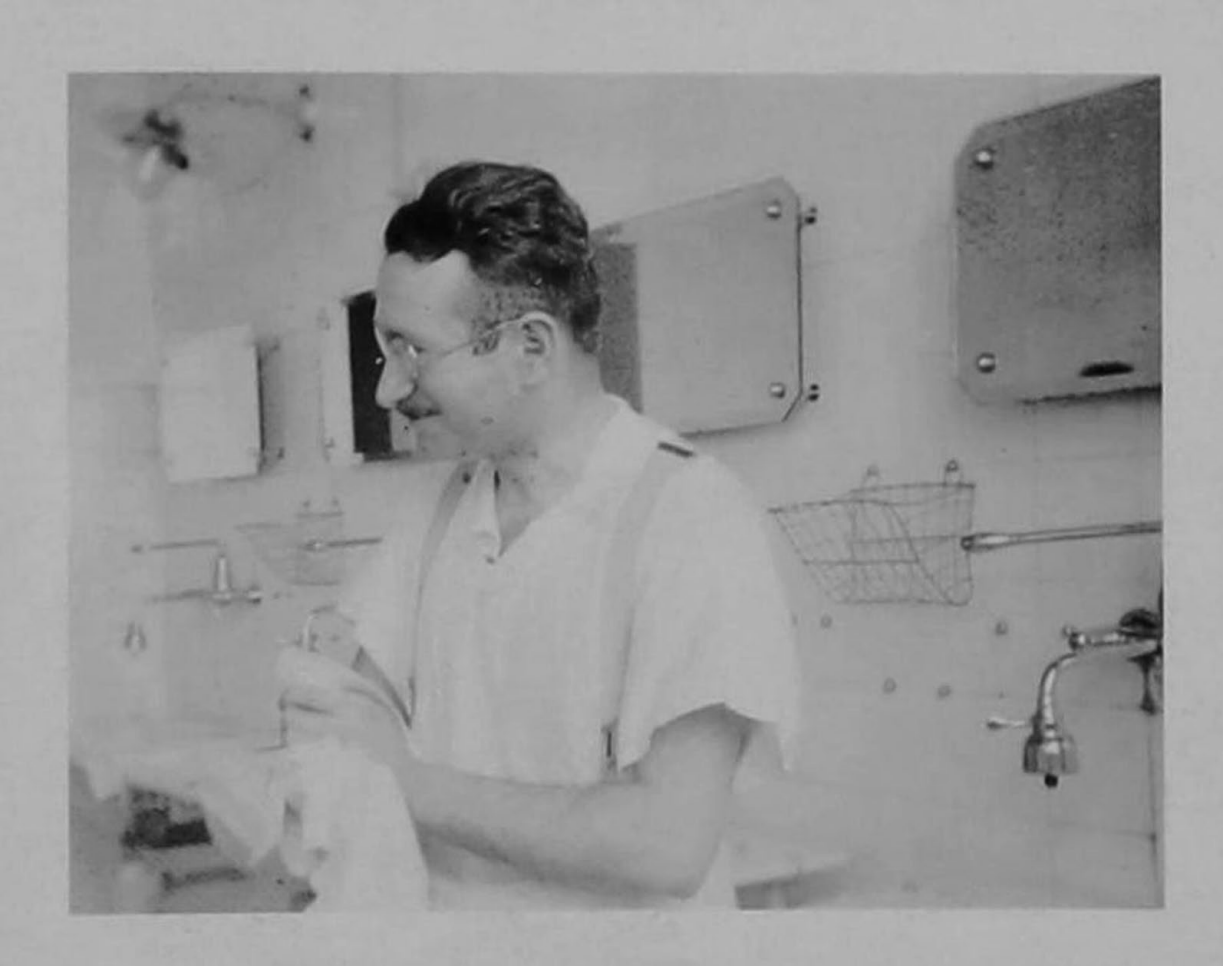
Ludwig Guttmann at a younger age. Undated photography, photographer unknown. *Source*: Wellcome Collection, Ludwig Guttmann archive collection. With permission

Guttmann hailed from Upper Silesia and grew up in a Jewish Orthodox home. After studying medicine and obtaining his doctorate in 1924, he became assistant to Otfrid Foerster (1873–1941) at the Neurological Clinic in Breslau (Wrocław) [55–57]. Foerster was a highly respected scientist at the time, attracting many physicians from abroad, including subsequently famous names such as Wilder Penfield and Percival Bailey [58]. Guttmann received a thorough training in clinical neurology and rehabilitation methods, and also learned various neurosurgical techniques and scientific methodology [59] (Fig. 4). At Foerster’s suggestion, he became familiar with the technique of pneumencephalography, a relatively new diagnostic procedure at the time. After a 1-year stay in Hamburg, Guttmann returned to Breslau as a senior physician in 1928, where he received his teaching license (*Habilitation*) in 1930. In those years he was also a member of the Society of German Neurologists or GDN, the predecessor organization of today’s DGN [60].

Soon after Hitler seized power, Guttmann was dismissed on June 30, 1933. Such measures were founded on the “Reconstitution Law” (“Law for the Restoration of the Professional Civil Service”, *Berufsbeamtengesetz*), which sanctioned the immediate removal of undesirable civil servants, especially those classified as “non-Aryan” [61]. How the Breslau clinic, with its allegedly friendly behavior towards Jews, and Guttmann himself, were viewed by the new rulers can easily be deduced from a statement by the loyal neurologist Walter Jacobi (1889–1938) that he sent to the Reich Physician Leader (*Reichsärzteführer*) Gerhard Wagner (1888–1939) in December of the same year:

“The ample ‘Jewification’ of our profession may be one explanation for the high percentage of Jewish physicians in the Foerster clinic; however, one should also take into account that Aryans, once employed, had to cope with Foerster’s authoritarian and capricious manner, as well as with a Jewish regime wielded in the clinic by Guttmann not long before the national uprising, a regime they couldn’t endure” (quoted from [48], S. 83).

After his dismissal, Guttmann, being a “Jew”, had no other choice but to work at a Jewish institution. Therefore, in July 1933, he took up a post as director of an Outpatient Clinic for Nervous Diseases founded especially for him at the Israelite Hospital in Breslau, which did not prevent him from pursuing the publication of notable papers [62–64]. He is said to have turned down several job offers from the United States and Portugal because, like many others, he could not imagine the new regime would last very long. Even before the Jewish pogroms on November 9, 1938 (*Reichskristallnacht* or “night of broken glass”), when the synagogue in Breslau was set on fire and Guttmann admitted to protecting Jews who sought shelter in the hospital, he had begun to consider the possibility of emigration to England; accordingly, a request for help, supported by Otto Schiff (1875–1952), the president of the German-Jewish Aid Committee, was sent to the Society for the Protection of Science and Learning.

On March 4, 1939, the Guttmanns and their two children left home with few meager belongings and 40 Reichsmarks in their pockets (only 10 Reichsmarks per person were allowed). In Oxford, Guttmann was granted a fellowship at the research laboratory of the Nuffield Department of Surgery, initially limited to six months, and endowed with 250 pounds per year. He was the fifth refugee the institution employed. Accordingly, the first years in Great Britain presented a financial hardship for the family. At the end of 1943, Guttmann became head of a department for spinal cord injuries, established at Stoke Mandeville Hospital in Aylesbury, 40 km east of Oxford, in preparation for the Allied landing in Normandy [65]. In his new capacity, he devoted himself to spinal column injuries and became a world-renowned authority in the treatment of paraplegics [66]. Having initiated the “Stoke Mandeville Games” he promoted the idea of the Paralympic Games, and the first ones were held in 1960. In 1945 he became a naturalized British citizen, and in 1966 was knighted. For his commitment he received numerous international awards, including the Grand Cross of Merit with Star (*Bundesverdienstkreuz mit Stern)* of the Federal Republic of Germany and honorary membership in the DGN in 1971.

The manner in which Guttmann was forced to emigrate was typical of many others like him. Fired only because of his categorization as a “Jew”, he was forced into marginalized occupations reserved for Jewish doctors. He also hesitated to leave for too long, hoping for a foreseeable end of Nazi rule. He made his decision to emigrate rather late, only a few months before the outbreak of war. Yet he was among the few who were able to pursue careers in their specialty while working abroad.

Aside from Guttmann, Albrecht Bethe is another DGN honorary member who was persecuted by the Nazi regime. Because his wife was considered “half-Jewish” under the Nuremberg Laws, he lost his professorship and was banned from his profession. However, he regained his academic position after the end of the war and was even appointed provost at the behest of the U.S. authorities [7]. At the age of 80, the DGN awarded him with an honorary membership. Walther Birkmayer, however, must be viewed as both perpetrator and “victim”: He was one of the staunch early supporters of the Nazi “movement” in Austria and worked from 1938 in the Racial Policy Office of the *Gauleitung* Vienna. When his grandmother was classified as “non-Aryan,” however, he was obliged to give up his Party and university posts and served henceforth as a military doctor in the Wehrmacht [19].

## Discussion

The results of our study highlight the comparatively rapid (self-)Nazification of German neurology before 1945, along with the very hesitant distancing from its own past after 1950 [67]. Among the 27 German and Austrian honorary members appointed to the DGN up to 1982, no less than 15 (56%) had been members of the NSDAP. This high percentage suggests that previous Party membership was neither a negative criterion for selection nor a limiting factor for appointment. One could even argue that it was exactly the other way around, because the percentage of former NSDAP members among honorary members of the DGN was higher than that in the medical profession overall (45%) [68]. If one includes membership in the SA and SS, 17 out of the 27 honorary members (63%) had at least formal contacts with the National Socialist state. Recent studies on the German Society for Pathology [69] and on dental associations [70] have found almost identical numerical ratios; recently published studies in relation to the German Society for Internal Medicine [71] and the psychiatric association also contain analogous data [72, 73].

Yet, here again, the time period plays a role. During the appointment period from 1952–1956 (Cluster 1), one out of ten honorary memberships was awarded to an individual persecuted by the regime (Bethe), three of them to more or less “non-political” individuals (Henneberg, Hochstetter, and Müller) and four to individuals “ambivalent” in their behavior towards the regime (the Vogts, von Weizsäcker, and Kleist) [33, 74]. Only with Reichardt it is safe to assume ideological support of the National Socialist system, but with Schönborn it cannot be excluded. Regarding the seven appointments made in 1952, not a single former NSDAP Party member was considered, although Heinrich Pette (1887–1964), the most influential neurologist of the Nazi era in matters of professional politics, was chairman of the newly established DGN. Pette himself had joined the Party in 1933 and in the same year signed the “vow of allegiance of professors to Adolf Hitler and the National Socialist State,” [75]. There might be an inclination to praise him for having a fine political instinct which his successors lacked; besides Pette, in the office of Chairman Schaltenbrand (1879–1979), Vogel (1900–1979) and Behrend (1919-1996) were also Party members [21, 76, 77].

Regarding appointments from 1976–1982, the historical findings are exactly the opposite. Cluster 2 includes only three appointments to formally exonerated men (Kalm, Lange, and Pass). There remain 17 individuals of whom 12 were verified members of the NSDAP, seven of the SA, and four of the SS. These facts lead to the conclusion that even around 1980 when selecting potential honorary members, the DGN, its chairmen, and the Board of Directors paid no attention to their possible “Nazi past” nor scrutinized them for such associations. Over the decades, the DGN as a professional association apparently avoided investigating and discussing within its inner circle the involvement of prominent protagonists—though perhaps with two exceptions: Hallervorden and Spatz were not considered for honorary membership. This strategy was maintained until the end of the 1990s, when the last members of Nazi organizations, Seitelberger and Becker, were awarded the Wilhelm Erb Commemorative Coin [78] and the Erb Mug [79].

From a present-day perspective we need to ask, what can explain the astonishingly high number of National Socialist-incriminated honorary members affiliated with the DGN? Even in the academic world, awards are nearly always tied to their historical context. Their attributions occur within a complex interplay of those who bestow honors, those who are honored, and the respective public. Historical research has recently introduced the concept of “award monitoring regimes,” which describes a regulatory and value-based framework underlying the procedures of honoring and dishonoring individuals under specific social constellations. This framework and these regimes, however, are strongly dependent on a contemporary point of view and are susceptible to change [80].

The research approach applied to the practice of the DGN in conferring honorary memberships provides notable insights. In the Federal Republic of Germany it was a common defense strategy in the 1950s to defend membership in the NSDAP or other Nazi organizations as a necessary evil—an evil that was accommodated to avoid the disadvantages that resulted from threatening potentially identifiable opponents of the system. At the same time, medical associations favored the narrative that only a few doctors—the number 350 was circulated—had been involved in the medical crimes of National Socialism [81]. The answer to the question “Who was a Nazi”, posed at the time by the West German medical societies, was: “almost nobody.” After denazification had passed into German hands, the answer to the same question by the so-called special tribunals (*Spruchkammern*) gradually turned out to be even more lenient [82]. As shown above by the example of Gustav Bodechtel, judgments were usually followed by successful appeals. The respective trials were made a mockery of when even members of the SA and SS were classified as “followers” and as “exonerated”; a status that applies especially to honorary members of the DGN, like Jacob, Zülch [40], and Bauer [83]. Hence the 1950s were shaped by a process of generous amnesty and reintegration of former Nazi supporters, which, when it concerned the past, was accompanied by a “consensus of silence,” leading to a continuation of elites. Thus, it must come as a surprise that between 1952 and 1956 the DGN did not award more honorary memberships to former Nazi offenders.

In the 1960s and 1970s, criticism of the approach “to put an end to it once and for all” gained momentum in West Germany. It was the time of the Auschwitz trials and public debates that addressed the killing of patients by the Nazi regime. However, many still turned a blind eye to the role of the professional medical societies during the Nazi era. Physicians active in professional politics stuck to the tactics of silence and ignoring questions when it came to the role of their institution during the Third Reich. Doctors who embraced the topic were branded as “individuals fouling their own nest,” and the involvement of their own profession in the crimes of National Socialism was denied. Two events in 1982 demonstrate in a startling way how this exact position was taken up by the German Neurological Society. First, a commemorative publication appeared in which the Nazi era was summarized under the heading “Years of Resistance.” Although this passage related to the resistance of neurologists against a forced merger with the Psychiatric Society in 1935, not a single word about the expulsion and murder of Jewish colleagues was included [84]. Second, the Society appointed five new honorary members, three of whom, Bauer, Birkmayer, and Seitelberger, had been members of the SS, and three others, Becker, Jacob, and Birkmayer, had been members of the SA. Except for Bauer, the others had also been members of the NSDAP. In this practice, the DGN was not alone; rather, it was a manifestation of the “common sense” of the time: The focus was on singular scientific achievements only, and any political implications considered taboo were avoided. But the fact that these distinctions are critically questioned today testifies to a radically altered historical awareness [85].

Among the honorary members presented in this paper, Ludwig Guttmann is the only one belonging to the large number of German and Austrian neurologists of Jewish descent who had to leave their home country after 1933 [67]. His honorary membership in the DGN is even more striking when compared to the fact that, among university lecturers in clinical disciplines, neurology and psychiatry had the highest proportion, at 65%, of those who were dismissed after 1933 [86]. In other words, there would certainly have been several other candidates qualified for honorary membership in terms of scientific accomplishments. There are primarily two reasons such candidates were not considered. First, as mentioned above, the DGN was not able or willing to face up to its own past. Second, most emigrants did not come back, were forgotten over the years, or simply flew “under the radar” of influential decision-makers within the Society’s inner circle. However, two Jewish scientists are listed among the international honorary members: in 1970 the French neuroradiologist Herman Fischgold (1899–1982) [87] and in 1971 the Romanian neurophysiologist and clinician Arthur Kreindler (1900–1988) [88]. It would seem obvious to interpret these later additions as a gesture of compensation; however, to date, this assumption lacks any evidence. It could also be dismissed as a footnote in history that Guttmann, who was persecuted by the Nazis, was granted by former SS-Captain Bauer during his tenure in 1971. Most likely Guttmann did not know about Bauer’s past. But this coincidence once again underlines the secondary role to which “the past” was relegated at that time. In other words: for decades, the awarding of honorary membership to the DGN depended on nothing but (scientific) reputation, and involvement in the National Socialist system was not taken into consideration.

## Conclusions: The German Neurological Society and its honorary members

Thus, the conclusions we can draw from our study are unambiguous. Following its foundation in 1950, the DGN made no effort to critically examine the implications of its honorary members and their role in the Third Reich. Clearly, moral integrity was practically ignored as a selection criterion. This cavalier attitude was maintained up to the 1990s. But such a stance also meant that the Jewish neurologists and neuroscientists expelled from their academic positions by the Nazi state were marginalized for a second time. While the historical and political awareness of the DGN has significantly changed over the years, the honorary members in question mentioned here, with links to the Nazi state, will not be deleted from the DGN website. However, the respective texts include a short biography with notes on the historical context and ethical issues [3]. In addition, the DGN has commissioned a further research project that focuses on the neurologists who were expelled and murdered during the Nazi era.

## Data Availability

Not applicable.

## Abbreviations

BRD: Bundesrepublik Deutschland (Federal Republic of Germany)
DDR: Deutsche Demokratische Republik (German Democratic Republic)
DGGN: Deutsche Gesellschaft für Geschichte der Nervenheilkunde (German Society for the History of the Neurosciences and Psychiatry)
DGN: Deutsche Gesellschaft für Neurologie (German Neurological Society)
DGP: Deutsche Gesellschaft für Pathologie (German Society for Pathology)
GDN: Gesellschaft Deutscher Nervenärzte (Society of German Neurologists)
KWIBR: Kaiser Wilhelm Institute for Brain Research
NS: National Socialist
NSDÄB: Nationalsozialistischer Deutscher Ärztebund (National Socialist German Doctors̕ League)
NSDAP: Nationalsozialistische Deutsche Arbeiterpartei (National Socialist German Workersʼ Party)
NSFK: Nationalsozialistisches Fliegerkorps (National Socialist Flying Corps)
NSLB: Nationalsozialistischer Lehrerbund (National Socialist German Lecturer Association)
SA: Sturmabteilung (Storm Troopers, “Brownshirts”)
SS: Schutzstaffel (“Nazi black shirts”)
U.S.: United States

## References

[1] Grond M, Thiekötter T (2016). Neurologen und Neurowissenschaftler in der NS-Zeit. Nervenarzt.

[2] Grond M, Thiekötter T (2020). Who was a Nazi? Neurologen und Neurowissenschaftler in der NS-Zeit. Nervenarzt.

[3] DGN/German Neurological Society. (2020). Verstorbene Ehrenmitglieder der Deutschen Gesellschaft für Neurologie. Retrieved March 3, 2022, from https://dgn.org/uber-uns/preise_ehrungen/

[4] Kumbier E (2009). Die Entstehung der Fachgesellschaften für Psychiatrie und Neurologie in der DDR. Schriftenreihe der DGGN.

[5] Martin M, Karenberg A, Fangerau H (2020). Männer ohne Vergangenheit. (Ehren)Vorsitzende der DGN nach 1957 und ihre NS-Belastung. Nervenarzt.

[6] Feichtinger J, Matis H, Sienell S, Uhl H (2013). Die Akademie der Wissenschaften in Wien 1938 bis 1945.

[7] Stahnisch FW (2016). From “nerve fiber regeneration” to “functional changes” in the human brain. On the paradigm-shifting work of the experimental physiologist Albrecht Bethe (1872–1954) in Frankfurt am Main. Frontiers in Systems Neuroscience.

[8] Leven K-H, Rauh P, Thum A, Ude-Koeller S (2018). Die Medizinische Fakultät der Friedrich-Alexander-Universität Erlangen-Nürnberg. Kontexte – Köpfe – Kontroversen.

[9] Eisenberg U (2016). Hirnschwellung, Hirnstamm, Hirngespinste? Der Würzburger Neurologe und Psychiater Martin Reichardt (1874–1966) und sein Verhältnis zum Nationalsozialismus. Schriftenreihe der DGGN.

[10] Satzinger H (1998). Die Geschichte der genetisch orientierten Hirnforschung von Cécile und Oskar Vogt in der Zeit von 1895 bis ca. 1927.

[11] Satzinger H (1999). Krankheiten als Rassen. Politische und wissenschaftliche Dimensionen eines biomedizinsichen Forschungsprogramms von Cécile und Oskar Vogt zwischen Tiflis und Berlin (1919–1939). Medizinhistorisches Journal.

[12] Benzenhöfer U (2007). Der Arztphilosoph Viktor von Weizsäcker. Leben und Werk im Überblick.

[13] Martin M, Fangerau H, Karenberg A (2020). Zu viel der Ehre? Ehrenmitglieder der DGN von 1954 bis 1982. Der Nervenarzt.

[14] Holdorff B (2019). Die Neurologie in Berlin 1840–1945. Aufstieg und Niedergang.

[15] Satzinger, H. (2014). Cécile Vogt (1875–1962). In *Encyclopedia of life sciences*. 10.1002/9780470015902.a0025071

[16] Hansson N, Palmen L, Padrini G, Karenberg A (2020). Babinski, Bektherev, Cerletti, Head, Hitzig: European Neurologists nominated for the Nobel Prize 1901–1950. European Neurology.

[17] Charité – Universitätsmedizin Berlin (2020). Ärztinnen im Kaiserreich. Wo bleiben die Frauen in der Medizingeschichte? Retrieved April 14, 2020, from https://geschichte.charite.de/aeik/index.html

[18] Bundesarchiv Berlin. NSDAP-Gaukartei, R9361-X KARTEI/41220754. Franz Seitelberger, No. 8121419.

[19] Czech H, Zeidman LA (2014). Walther Birkmayer, co-describer of L-DOPA, and his Nazi connections: Victim or perpetrator. Journal of the History of the Neurosciences.

[20] Zeidman LA, Kondziella D (2014). Peter Becker and his nazi past: The man behind Becker muscular dystrophy and Becker myotonia. Journal of Child Neurology.

[21] Karenberg A, Martin M, Fangerau H (2020). Neurologen und Neurowissenschaftler in der NS-Zeit: Versuch einer Bewertung. Der Nervenarzt.

[22] Peiffer J (1997). Hirnforschung im Zwielicht. Beispiele verführbarer Wissenschaft aus der Zeit des Nationalsozialismus. Julius Hallervorden – J. J. Scherer – Berthold Ostertag.

[23] Peiffer J, Kaufmann D (2000). Neuropathologische Forschung an “Euthanansie”-Opfern in zwei Kaiser-Wilhelm-Instituten. Geschichte der Kaiser-Wilhelm-Gesellschaft im Nationalsozialismus. Bestandsaufnahme und Perspektiven der Forschung.

[24] Tackenberg B, Oertel WH, Hippius H, Holdorff B, Schliack H (2006). Hans Jacob. Nervenärzte 2.

[25] Zeidman LA (2017). Hans Jacob and brain research on Hamburg “euthanasia” victims: “Awaiting further brains!”. Neurology.

[26] Mennel H-D (2014). Hans Jacob und Klaus Joachim Zülch als Vertreter einer morphologischen Nervenheilkunde. Schriftenreihe der DGGN.

[27] Topp S, Beddies T, Hübener K (2004). Der “Reichsausschuß zur wissenschaftlichen Erfassung erb- und anlagebedingter schwerer Leiden”. Zur Organisation und Ermordung minderjähriger Kranker im Nationalsozialismus 1939–1945. Kinder in der NS-Psychiatrie.

[28] Peiffer J (2005). Wissenschaftliches Streben als Tötungsmotiv? Zur Kennzeichnung von Opfern auf deren Krankenakten und zur Organisation und Unterscheidung von Kinder-“Euthanasie” und T4-Aktion.

[29] Burlon, M. (2009). *Die "Euthanasie" an Kindern während des Nationalsozialismus in den zwei Hamburger Kinderfachabteilungen*. Med. Thesis.

[30] Peiffer J (1999). Assessing the neuropathological research carried out on victims of the “euthanasia” programme. Medizinhistorisches Journal.

[31] Czech, H. (2001). Forschen ohne Skrupel. Die wissenschaftliche Verwertung von Opfern der NS-Psychiatriemorde in Wien. Resource document. eForum zeitgeschichte, 1. Retrieved February 21, 2019, from http://www.eforum-zeitgeschichte.at/frameseta3.htm

[32] Beddies, T., & Hübener, K. (2003). Kinder und Jugendliche in der brandenburgischen Heil- und Pflegeanstalt Görden als Opfer der NS-Verbrechen. In K. Hübener, & M. Heinze (Eds.), *Brandenburgische Heil- und Pflegeanstalten während der NS-Zeit* (pp. 129–154). Be.bra (Schriftenreihe zur Medizin-Geschichte des Landes Brandenburg, 3).

[33] Kondziella D (2009). Thirty neurological eponyms associated with the Nazi era. European Neurology.

[34] Vollnhals C (1991). Entnazifizierung. Politische Säuberung und Rehabilitierung in den vier Besatzungszonen 1945–1949.

[35] Bundesarchiv Berlin. NSDAP-Mitgliederkartei, R 9361-IX Kartei. Gustav Bodechtel, No. 3400838.

[36] Landesarchiv Nordrhein-Westfalen. Gustav Bodechtel, Entnazifizierungsakte, NW 1002 MED Nr. 01458.

[37] Sachse C, Weisbrod B (2002). “Persilscheinkultur”. Zum Umgang mit der NS-Vergangenheit in der Kaiser-Wilhelm/Max-Planck-Gesellschaft. Akademische Vergangenheitspolitik. Beiträge zur Wissenschaftskultur der Nachkriegszeit.

[38] Landesarchiv Nordrhein-Westfalen. Gustav Bodechtel, Personalakte BR-Pe Nr. 2546.

[39] Baumann T, Sparing F, Martin M, Fangerau H (2020). Neurophysiologen im Nationalsozialismus – Hans Berger, Paul Hoffmann, Richard Jung und Alois E. Kornmüller. Klinische Neurophysiologie.

[40] Martin M, Fangerau H, Karenberg A (2020). Die zwei Lebensläufe des Klaus Joachim Zülch. Der Nervenarzt.

[41] Staatsarchiv Hamburg. Entnazifizierungsakte Hans-Robert Müller.

[42] Mitscherlich A, Mielke F (1962). Medizin ohne Menschlichkeit: Dokumente des Nürnberger Ärzteprozesses.

[43] Benzenhöfer U (2012). Die Frankfurter Universitätsmedizin zwischen 1933 und 1945.

[44] Neumärker K-J, Bartsch AJ (2002). Karl Kleist (1879–1960): A pioneer of neuropsychiatry. History of Psychiatry.

[45] Kaendler S, Volk S, Sachunsky I, Pflug B, Beckmann H, Neumärker K-J (1995). Karl Kleist – his attitude and point of view on National Socialist politics – 1933–1945. Endogenous Psychoses. Leonhard’s Impact on Modern Psychiatry.

[46] Koch G (1993). Humangenetik und Neuro-Psychiatrie in meiner Zeit (1932–1978). Jahre der Entscheidung.

[47] Kranz H, Pongratz LJ (1977). Heinrich Kranz. Psychiatrie in Selbstdarstellungen.

[48] Schmuhl H-W (2016). Die Gesellschaft Deutscher Neurologen und Psychiater im Nationalsozialismus.

[49] Klee E (2013). Das Personenlexikon zum Dritten Reich. Wer war was vor und nach 1945.

[50] Martin M, Karenberg A, Fangerau H (2016). Neurologie und Neurologen in der NS-Zeit: Hirnforschung und “Euthanasie”. Der Nervenarzt.

[51] Alexander, L. (1945) Combined Intelligence Objectives Sub-committee (CIOS) report. Public mental health practices in Germany: Sterilization and execution of patients suffering from nervous or mental disease. Item No. 24. File No. XXVIII-50.

[52] Goldenberg G (2011). Karl Kleist: A Nazi behind the map?. Cortex.

[53] Martin M, Karenberg A, Fangerau H (2020). Zwischen “Affirmation und Kritik”: Karl Kleist und Viktor von Weizsäcker zwischen 1933 und 1945. Der Nervenarzt.

[54] Hagner M, Schmuhl H-W (2003). Im Pantheon der Gehirne. Die Elitegehirnforschung bei Oskar und Cécile Vogt. Rassenforschung am Kaiser-Wilhelm-Instituten vor und nach 1933.

[55] Silver JR (2013). The making of Ludwig Guttmann. Journal of Medical Biography.

[56] Stahnisch FW, Tynedal JD (2012). Sir Ludwig Guttmann (1899–1980). Journal of Neurology.

[57] Akkermans R (2016). Ludwig Guttmann. The Lancet Neurology.

[58] Martin M, Karenberg A, Fangerau H (2020). Die ambivalente Haltung Otfrid Foersters (1973–1941) gegenüber dem Nationalsozialismus. Der Nervenarzt.

[59] Dubinski D, Collmann H, Eisenberg U, Collmann H, Dubinski D (2017). Sir Ludwig Guttmann (1899–1980). Verraten – Vertrieben – Vergessen. Werk und Schicksal nach 1933 verfolgter deutscher Hirnchirurgen.

[60] Gesellschaft deutscher Nervenärzte (1929–1933). Mitgliederverzeichnisse. In *Verhandlungen der Gesellschaft deutscher Nervenärzte*. F. C. W. Vogel.

[61] Martin M, Karenberg A, Fangerau H (2016). Neurologie und Neurologen in der NS-Zeit: Voraussetzungen und Rahmenbedingungen vor und nach 1933. Der Nervenarzt.

[62] Guttmann L, Bumke O, Foerster O (1936). Physiologie und Pathologuie der Liquormechanik und Liquordynamik. Handbuch der Neurologie.

[63] Guttmann L, Bumke O, Foerster O (1936). Röntgendiagnostik des Gehirns und Rückenmarks durch Kontrastverfahren. Handbuch der Neurologie.

[64] Silver JR (2001). Sir Ludwig Guttmann’s publications under the Nazis. Spinal Cord.

[65] Silver JR, Weiner M-F (2013). Sir Ludwig Guttmann: His neurology research and his role in the treatment of peripheral nerve injuries. Journal of the Royal College of Physicians of Edinburgh.

[66] Goodman S (1986). Spirit of stoke Mandeville. The story of Sir Ludwig Guttmann.

[67] Zeidman LA (2020). Brain Science under the Swastika: Ethical violations, resistance, and victimization of neuroscientists in Nazi Europe.

[68] Rüther M, Jütte R (1997). Ärztliches Standeswesen im Nationalsozialismus 1933–1945. Geschichte der deutschen Ärzteschaft. Organisierte Berufs- und Gesundheitspolitik im 19. und 20. Jahrhundert.

[69] Schmidt M, Gräf C, Groß D (2020). Virchow-Preisträger und Ehrenmitglieder der DGP und ihr Verhältnis zum Nationalsozialismus. Eine Querschnittsstudie. Der Pathologe.

[70] Wilms KF, Groß D (2021). Blind in the right eye? The practice of awarding honorary memberships by German and Austrian dental societies to Nazi dentists (1949–1993): A study on the role of National Socialism in post-war dentistry. Endeavour.

[71] Forsbach R, Hofer H-G (2018). Internisten in Diktatur und junger Demokratie. Die Deutsche Gesellschaft für Innere Medizin 1933–1970.

[72] DGPPN/German Association of Psychiatry, Psychotheapy and Psychosomatics. (2020). Ehrenmitglieder. Retrieved June 11, 2020, from https://www.dgppn.de/die-dgppn/ehrungen-und-preise/ehrungen.html

[73] Dörre S (2021). Zwischen NS-"Euthanasie" und Reformaufbruch. Die psychiatrischen Fachgesellschaften im geteilten Deutschland.

[74] Zeidman LA (2011). Neuroscience in Nazi Europe part I: Eugenics, human experimentation, and mass murder. Canadian Journal Neurological Sciences.

[75] Martin M, Karenberg A, Fangerau H (2020). Heinrich Pette (1887–1964) und die schwierige Bewertung seiner Rolle von der Weimarer Republik bis in die BRD. Der Nervenarzt.

[76] Collmann H (2008). Georges Schaltenbrand (26.11.1897–24.10.1979). Würzburger Medizinhistorische Mitteilungen.

[77] Martin M, Karenberg A, Fangerau H (2020). “… voll und ganz auf dem Boden des Nationalsozialismus“? Paul Vogel (1900–1979). Der Nervenarzt.

[78] DGN/German Neurological Society. (2020). Awards – Wilhelm Erb-Gedenkmünze. Retrieved November 26, 2020, from https://dgn.org/uber-uns/awards

[79] DGN/German Society for Neurology. (2020). Awards – Erb-Becher. Retrieved November 26, 2020, from https://dgn.org/uber-uns/awards/

[80] von Reeken D, Thießen M (2016). Ehrregime. Akteure, Praktiken und Medien lokaler Ehrungen in der Moderne.

[81] Jachertz N, Jütte R (1997). Phasen der “Vergangenheitsbewältigung” in der deutschen Ärzteschaft nach dem Zweiten Weltkrieg. Geschichte der deutschen Ärzteschaft. Organisierte Berufs- und Gesundheitspolitik im 19. und 20. Jahrhundert.

[82] Fürstenau J (1969). Entnazifizierung. Ein Kapitel deutscher Nachkriegspolitik.

[83] Schmidt M, Westemeier J, Groß D (2019). Renowned MS specialist and National Socialist: The two lives of neurologist Helmut J. Bauer (1914–2008). Neurology.

[84] Seitz D (1982). 75 Jahre Deutsche Gesellschaft für Neurologie 1907–1982.

[85] DGN/German Society for Neurology. (2020). Stellungnahme zur NS-Geschichte der DGN, 21 September 2020. Retrieved November 26, 2020, from https://dgn.org/neuronews/neuronews/stellungnahme-zur-ns-geschichte-der-dgn/

[86] Kröner H-P (1989). Die Emigration deutschsprachiger Mediziner im Nationalsozialismus. Berichte zur Wissenschaftsgeschichte.

[87] Nahum H (2008). L'éviction des médecins juifs dans la France de Vichy. Archives Juives.

[88] Sîrbu C-A (2006). Arthur Kreindler (1900–1988). Journal of Neurology.

